# The effectiveness of introducing Group Prenatal Care (GPC) in selected health facilities in a district of Bangladesh: study protocol

**DOI:** 10.1186/s12884-017-1227-6

**Published:** 2017-01-31

**Authors:** Marufa Sultana, Rashidul Alam Mahumud, Nausad Ali, Sayem Ahmed, Ziaul Islam, Jahangir A. M. Khan, Abdur Razzaque Sarker

**Affiliations:** 1 Health Economics and Financing Research, International Centre for Diarrhoeal Disease Research, Bangladesh (icddr,b), 68 Shaheed Tajuddin Ahmed Sharani, Mohakhali, Dhaka 1212 Bangladesh; 2 Liverpool School of Tropical Medicine, Pembroke Place, Liverpool, UK; 3 Karolinska Institutet, Stockholm, Sweden; 4 University of Strathclyde, Glasgow, Scotland

**Keywords:** Group prenatal care, Pregnant women, ANC, PNC

## Abstract

**Background:**

Despite high rates of antenatal care and relatively good access to health facilities, maternal and neonatal mortality remain high in Bangladesh. There is an immediate need for implementation of evidence-based, cost-effective interventions to improve maternal and neonatal health outcomes. The aim of the study is to assess the effect of the intervention namely Group Prenatal Care (GPC) on utilization of standard number of antenatal care, post natal care including skilled birth attendance and institutional deliveries instead of usual care.

**Methods:**

The study is quasi-experimental in design. We aim to recruit 576 pregnant women (288 interventions and 288 comparisons) less than 20 weeks of gestational age. The intervention will be delivered over around 6 months. The outcome measure is the difference in maternal service coverage including ANC and PNC coverage, skilled birth attendance and institutional deliveries between the intervention and comparison group.

**Discussion:**

Findings from the research will contribute to improve maternal and newborn outcome in our existing health system. Findings of the research can be used for planning a new strategy and improving the health outcome for Bangladeshi women. Finally addressing the maternal health goal, this study is able to contribute to strengthening health system.

## Background

There is growing concern about prenatal care for women especially in developing countries, where 80% of the world’s women live [1]. Approximately ninety-nine percent of maternal and neonatal deaths occur in developing countries which is higher in rural areas and among poorer communities [2]. There is an immediate need for implementation of evidence-based, cost-effective interventions to improve maternal and neonatal health outcomes [3]. The Government of Bangladesh is committed to achieving Millennium Development Goal (MDG) 4 and 5; to reduce the under-5 mortality ratio from 146 per 1000 live births in 1990 to 48 per 1000 live births in 2015 and to improve maternal health, by reducing the maternal mortality ratio from 574 to 143 deaths per 100.000 live births by 2015 respectively.

However, the antenatal care (ANC) and postnatal care (PNC) received by medically trained provider is still very low and around 55 and 27% respectively [4]. Although the current practice of the safe motherhood programmes in Bangladesh is to advice pregnant women to deliver their babies in facilities but almost 62% are performed at home (BDHS 2014). Further, though breastfeeding initiation within one hour after birth has increased over time, still it remains below 50% [5]. Despite a fair amount of advocacy around maternal and newborn care, real progress on the ground remains slow [6]. Further efforts are needed to ensure better coverage of antenatal care, as well as improved quality and content of this care [7].

Major causes of maternal death are largely haemorrhage (31%), eclampsia (20%), and abortion (15%) in Bangladesh [5, 7]. Pregnant women are advised to seek care from a medically trained provider or from a facility when they experience complications during pregnancy, at delivery, or postpartum period till 42 days. There are some BCC activities on birth planning and certain danger signs that women may encounter during pregnancy, at delivery, or during postpartum period but the progress is still slow.

The health care that a woman receives during pregnancy, at the time of delivery, and soon after delivery is important for the survival and well-being of both the mother and the child. Prenatal care is a type of preventive care that provides regular check-ups that allow doctors or midwives to treat and prevent potential health problems throughout the course of the pregnancy while promoting healthy lifestyle that benefit both mother and child [8]. Access to prenatal care is critical to achieving the MDGs yet only 55% pregnant woman received prenatal care in Bangladesh in 2009–2013 [4]. In Bangladesh, generally pregnant women receive prenatal care individually by a provider in a private examination room in public, private or NGOs facilities. A previous study found that, traditional individual prenatal care can be supplemented by group prenatal classes, which facilitate support networks, social interaction, and additional education [9]. Group prenatal ideally suited for mothers in developing countries where adverse cultural and traditional practices and low-quality health services interfere with satisfactory implementation of prenatal care [10, 11].

Meeting in a group setting with other women of the same gestational age who are experiencing similar physiological changes of pregnancy nurtures supportive relationships among them are less common in Bangladesh. In the United State, Group prenatal care has been implemented successfully and has had positive impact on perinatal outcomes [12–14]. It provides an integrated approach to prenatal care in a group setting, incorporating family members, peer support, and education. It empowers women by valuing the knowledge and experience that each woman brings to the group, and increases this knowledge through skills building and education. The positive influence of group prenatal care has been strongest for women with low socioeconomic status which not been tried much in a resource poor country like Bangladesh. It is also found that group prenatal care has to positively influence women’s health outcomes after pregnancy and to improve the utilization rate of preventive health services [15]. In Bangladesh, a programme for improving maternal, neonatal and child health in the urban slums named MANOSHI by BRAC also successfully organized group sessions among the women with third trimester of pregnancy for giving health education and encouraging the mother for making a birth plan, safe and hygienic delivery, neonatal and postnatal care [16]. In this project we intend to introduce the GPC in public health facility instead of existing prenatal care. In this Group Prenatal Care (GPC) intervention, prenatal assessment, education and skills development, facilitates learning will be incorporated that will encourage knowledge exchange, and will develop mutual support from its group members which may improve maternal and neonatal outcome.

## Aims and objectives

The overall goal of the project intends to introduce a Group Prenatal Care (GPC) model in selected healthcare facilities in Bangladesh. The specific objective of the study is to assess the effectiveness of GPC in change in coverage of standard number of ANC, PNC, including skilled birth attendance and institutional deliveries; the impact of GPC on maternal and neonatal outcome like preterm birth, caesarean delivery, low birth weight, postpartum complications and breastfeeding rate; and to estimate the incremental cost-outcome ratio of introducing Group Prenatal care in selected facilities. A qualitative study will also be conducted to explore the acceptability and perception of the new GPC model among the intervened mothers.

## Conceptual framework

**Fig. 1:**
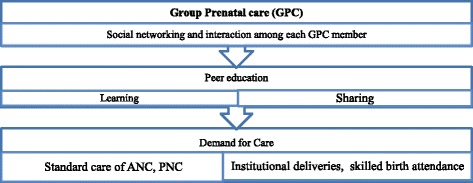
Conceptual framework

In group prenatal care, prenatal assessment, education and skills development will be occurred in an atmosphere that facilitates learning, encourages free exchange, and develops mutual support [17]. Group care will permit more time for pregnant women to develop a relationship among each other and to discuss recommended content of care. Peer education approaching (i.e. through peer participation) maternal health promotion will be carried out through peer educators like current pregnant women and experienced mothers. It is expected that group care with peer education will increase interaction with each other of the group members that can encourages women to utilize their knowledge through skill building which may improve their recommended healthcare utilization throughout the pregnancy period. Women thus are better able to take control of their adverse health situation during prenatal and postnatal period Fig. 1.

## Research design and methods

The study is quasi-experimental in design. The participants who are joining the GPC will be compared with those who receive standard individual prenatal care. Those who are will join the intervention program considered as “intervention group”. On the contrary, ‘comparison group’ are those who do not join this intervention. The intervention group will receive group prenatal care, and the comparison group will receive existing individual prenatal care as provided by the facilities following guidelines recommended by the Ministry of Health and Family Welfare (MOHFW), Government of the People’s Republic of Bangladesh.

### Inclusion criteria for participants

Potential participants for either group are to be pregnant women with less than 20 weeks of gestational age, between the age of 18 and 42, without medical complications like pregnancy with jaundice, heart disease, asthma, diabetes, and willing to participate in this study.

### Exclusion criteria for participants

Pregnant women with more than 20 weeks of gestational age and with medical conditions requiring special care are excluded from the study.

### Inclusion criteria for health centers

The health centers will be selected based on two inclusion criteria: at least 10–15 number of clients for ANC visits per day and availability of at least one trained healthcare provider in the facility.

## Sample size

The sample size is estimated using the technique proposed by [18, 19] for comparing two independent proportions. To calculate the sample sizes for detecting difference between intervention and comparison groups, following formula has been employed

$$ \mathrm{n}=\frac{{\left[\ {\mathrm{Z}}_{\upalpha}\sqrt{\left(\mathrm{r}+1\right)\overline{\mathrm{P}}\overline{\mathrm{Q}}}+{\mathrm{Z}}_{\upbeta}\sqrt{\Big({\mathrm{r}\mathrm{P}}_1{\mathrm{Q}}_1+{\overline{\mathrm{P}}}_2{\overline{\mathrm{Q}}}_2}\ \right]}^2}{{\mathrm{r}\updelta}^2} $$

Where,

a) $$ \updelta =\left|{\mathrm{P}}_2-{\mathrm{P}}_1\right| $$, b) $$ \overline{\mathrm{P}}=\frac{{\mathrm{P}}_1+\mathrm{r}{\mathrm{P}}_2}{\mathrm{r}+1} $$, c) $$ \overline{\;\mathrm{Q}}=1-\overline{\mathrm{P}} $$

d) α, the probability of type I error,e) β, the probability of type II error, or (1-power of the test)

f) P_1_, proportion of characteristic present in intervention group, g) P_2_, proportion of characteristic present in comparison group d) r, ratio of required sample size from two groups (*r* = 1 in this study)

It is expected that due to intervention different types of indicators (Table 1) will change. Using this percentage of expected change difference in intervention and comparison groups at 5% error level and 80% power the estimated sample size for each group is reported in following table. The highest sample size (250) is estimated for breastfeeding rate. Considering the 15% lost to follow-up, the sample size is 288 for each group. Therefore, in total 576 pregnant women (288 cases and 288 comparisons) will be enrolled randomly in this study. Recruitment will take place from May 20, 2015 to May 30, 2016 and intervention was completed on October 30, 2016. Data collection is ongoing now among the enrollees.

**Table 1:** Sample size estimation

Indicators	Parentage	% of expected change	Required sample size	Sample size (15% lost to follow-up rate)	Total sample
ANC^1^	55.0%	14%	202	232	464
PNC^1^	27.0%	15%	170	196	392
Skilled-birth attendance^1^	31.7%	16%	158	182	364
Institutional deliveries^1^	28.8%	13%	227	261	522
Preterm birth^2^	22.30%	−12%	164	189	378
Low birth weight^3^	36%	−12%	245	282	564
Postpartum complications	36%	−13%	208	239	478
Breastfeeding rate (2–3 m)^1^	71.0%	11%	250	288	576

## Randomization

Participants are randomly assigned to the intervention and comparison groups. After meeting the inclusion criterion and obtaining informed consent block randomization is used. Computer-generated random numbers is assigned which have been pre-assigned to one of the two groups (intervention and comparison). The random numbers corresponding to either group has been sealed in an envelope numbered sequentially. The sealed envelope is opened after obtaining the informed consent. Until opening of the envelope, neither the participants nor the healthcare provider is aware of the group that they are assigned to. The envelope that contained “A” meaning “Intervention group” and the envelope contained “B” meaning “Comparison group”.

## Study site

There are substantial numbers of health care centres in any district level in Bangladesh provided by the government, non-governmental organization (NGOs), private providers etc. For this study a government running Maternal & Child welfare Centre (MCWC) is selected in Chandpur district. This municipality area of Chandpur has a population of 94,821 with 49.23% women population. Among the total registered pregnant women, 98% received 1st ANC but only 7% of pregnant women received 4th ANC from qualified health care provider in this municipal [20]. Intervention and comparison are selected in same health centre because it serves similar populations with regard to socio-demographic factors.

## Intervention

**Fig. 2:**
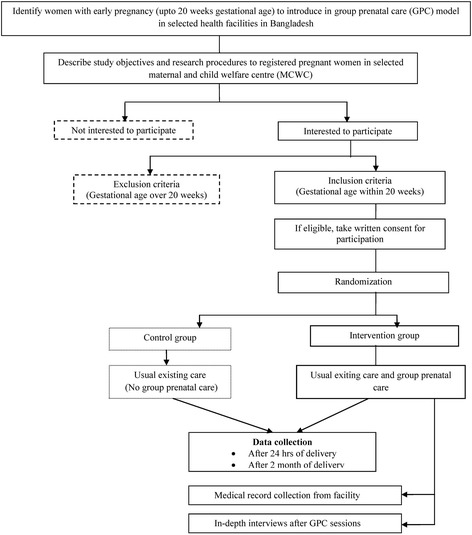
Intervention flow chart

Before designing the intervention a small piloting has carried out to observe how the group prenatal care (GPC) will be accepted in real field or not. The experience from the piloting has summarized and necessary adjustment of the intervention was carried out. Before starting the intervention, necessary trainings and workshops was conducted for the team members (group moderator, study nurse and support staffs) to improve their knowledge and skills and for making the common understanding about the GPC since skills of educators are the key of success of such intervention. After the completion of training and workshop, one or two discussion sessions has conducted for clearing their roles and their understanding about the GPC to find if there is any gap Fig. 2.

## Intervention flow chart

### Procedure to implement GPC model

Pregnant women in selected health centre are informed about the study at the booking visit or during registration. The study objectives and procedure of intervention explained clearly to the pregnant women and their husbands/guardians or accompanied persons by the project team. They informed about the content, description of the procedure and potential benefit for the enrolment of the study. Written informed consent was taken from the participants as well as their guardian prior to the study.

### Formation of group for the intervention

Each group consisted of 6 to 10 women. They met 4 to 6 times throughout the pregnancy period. Groups were formed considering the gestational age of the women. Each session continue up to two hours. The entire GPC sessions are held in separate rooms with presence of pregnant mothers of intervention group. It can reduce the likelihood of mixing with other pregnant mothers.

### Conduct sessions within group

The intervention consists of group sessions, providing BCC materials and regular follow-up to the participants. In each session starts after individual physical assessment of women by the qualified healthcare provider of the facility. The group discussions take 30–40 min and focuses sequentially on education and skills-building specific to prenatal and postnatal care with the help of study nurse. Session themes include the prenatal nutrition, common discomforts of pregnancy, caring of mother’s health during pregnancy, pregnancy danger signs, decisions of pregnancy care, developing a birth plan, breastfeeding, postpartum adjustment, medical procedures and tests during pregnancy and new baby care. In addition, key messages focused on maternal, neonatal and child health provides through leaflets to the intervention group.

### Follow up of the participants

Detailed information of registered women including address and phone number are taken for communication. Mobile follow-up is carried out regularly to track all of the participants.

## Cost of intervention

Incremental Cost i.e. the additional costs those will be used over the existing facilities for implementing group prenatal care intervention in these facilities will also be calculated. For calculating the incremental cost of introducing GPC, ingredient method will be applied which comprises, listing all types of inputs by activity and the quantities and prices for each input [21]. Both recurrent and capital cost will be included in the total cost. However, fixed cost and variable cost will be captured and presented separately. Capital cost will be annuitized and adjusted for inflation-adjusted discounting rate. With the discounting the present values of all costs will be calculated taking into account of the time when these costs will be incurred with their opportunity costs [22]. If the cost and outcome will take in different year, then 5% discount rate will be used.

Incremental cost-outcome ratio will be calculated i.e. if we switch one alternative to another alternative, the change in cost and outcome will be compared. Change in various effects between intervention and comparison group due to introducing GPC will be considered as the change in outcome/success of the intervention.

## Qualitative assessment

As the nature of the project is participatory, the qualitative evaluation will be conducted. The proposed study can obtain valuable information about the experience of the mother from GPC. Throughout the project period qualitative data collection such as conducting interview with mothers (in-depth interview) will be done to explore the perception and experience of mothers, expressed needs, health providers’ interactions and skills, institutional inadequacies, needs and benefits of women friendly GPC.

## Method of data collection

### Quantitative data

This study is prospective in nature and follow up of participants will be done from early pregnancy through 2 months postpartum. Both structured and semi-structured questionnaire will be used for data collection. Data will be collected at two points of time: 24 h after delivery, and 2 months after delivery. Cost data will be collected through structured and semi-structured questionnaire, observation checklist, hospital database and compilation of service statistics. Gestational age at birth, birth weight, neonate hospital stay, and maternal outcomes will be taken from medical records in hospitals and health centres, and individual structured interviews. Review of medical records and structured interviews will be carried out by trained study nurses.

### Qualitative data

The in-depth interview will be conducted with mothers to understand their expression, fill gaps in knowledge or to identify contextual factors that influenced impact of GPC. For this purposes, at least 6–8 in-depth interviews will be conducted in the study sites, however, actual number will be determined based on data saturation against explored board themes. In-depth interviews will be carried out by using flexible guideline. After consent, all interviews will be recorded by using digital recorder.

## Outcome of the study

### Primary outcome

Primary outcomes will include ANC and PNC coverage, skilled birth attendance and institutional deliveries, low birth weight (<2500 g), preterm birth, caesarean delivery, postpartum complications and breastfeeding rate (2 months postpartum). It is expected that due to GPC some outcome variables like ANC, PNC, skilled birth attendance, institutional deliveries and breastfeeding rate will be improved.

### Secondary outcomes

Some secondary outcomes like pregnancy induced hypertension, infant admission to hospital and perinatal death will be documented for both groups.

## Data analysis

The analysis of quantitative data will be based on a before and after assessment of the selected indicators, calculation of changes, and statistical significance based on 95% confidence intervals and other standard significance tests. Independent sample *t*-test for mean and proportion will be performed to test significance of difference in outcome variables between intervention and comparison group. To examine the normality of continuous outcome variable (like, low-birth-weight) histogram will be drawn. If the outcome variable found non-normally distributed, non-parametric test method (like, Kruskal–Wallis) will be employed. Advanced statistical techniques (such as multivariate regression analyses), will be used to document the net effects of the interventions on the outcome indicators after controlling for the individual-level socioeconomic factors. Data analysis will be done by using STATA 13.0.

For qualitative data analysis, content analysis will be done manually with qualitative information obtained through in-depth interviews. Immediate after the interview, the interviewers will complete the field-notes and transcribe the recording. After transcribing the interview, they will read the transcript carefully to identify potential themes. After then coding of data will begin through identifying possible themes and sub-themes.

## Discussion

A health care system aiming to reduce morbidity and mortality related to pregnancy must focus on maternal and newborn health. The World Health Organization recommended that “Ensuring skilled care for all women during pregnancy, childbirth, and the immediate postnatal period” is one of the strategies to reduce maternal and newborn mortality [23]. Methods described in this study could be a very innovative approach to implement GPC in existing selected facility and expected to have positive perinatal outcomes. Instead of providing usual care based on individual care, this group care at prenatal period will provide good understanding among mothers thus improvement of overall maternal and child health indicators is expected. Group care during pregnancy are proven to be useful in earlier studies that revealed group care ideally suited for mothers in developing countries and positive influence of GPC has been strongest for women with low socio-economic status [11, 12].

This study will compare birth and post partum outcomes for women who participated group prenatal care with women who received individual care. Results will be based on outcomes like increase utilization of prenatal and post natal care, institutional delivery, birth weight etc. Previous studies showed that Group prenatal care had statistically significant beneficial effects on gestational age and birth weight [24] and improve Utilization of postpartum family-planning services [25]. It is now imperative that we intend to experience this GPC approach on to exiting facility.

The study will contribute to improve maternal and newborn outcome in our existing health system. The intervention may improve the health well-being of both mother and child and the family as a whole. If successful, the project will scale up targeting more geographic areas and gradually cover both of the urban and rural areas for understanding the effectiveness of the group prenatal care. Findings of the research can be used for planning a new strategy and improving the health outcome for Bangladeshi women. Finally addressing the maternal health goal, this study is able to contribute to strengthening our health system.

## Abbreviations

ANC: Antenatal care
BCC: Behavioral Change Communication
ERC: Ethical Review Committee
GPC: Group prenatal care
icddr,b: International Centre for Diarrhoeal Disease Research, Bangladesh
LBW: Low-birth-weight
MCWC: Maternal & Child welfare Centre
MDG: Millennium Development Goal
MOHFW: Ministry of Health and Family Welfare
PNC: Post natal care
RRC: Research Review Committee
